# Supplementary material for: Content-rich biological network constructed by mining PubMed abstracts
Source: BMC Bioinformatics. 2004 Oct 8;5:147. doi: 10.1186/1471-2105-5-147 (PMC528731; doi:10.1186/1471-2105-5-147)
Supplement: Additional File 5 — The original Chilibot query results of the term "long-term potentiation (LTP)" and 22 other terms, limiting the latest references analyzed to the years 1990, 1995, 2000, and 2004. [file 1471-2105-5-147-S5.bz2 › chilibotAdditionalFile5/ltp2000/html/CREB_AMPA.html]

 


 **CREB** and **AMPA** 
  
Found 9 abstracts in PubMed,  **9 abstracts were retrieved and analyzed**.  


---

 Search Google  |
 PDF files only 
|  EDU domain only 

---

**Interactive relationship** (e.g. stimulation, inhibition, etc)

- However,  **AMPA**  receptor stimulation led to a calcium dependent phosphorylation of the nuclear transcription factor  **CREB** , which could be prevented by inhibitors of MEK or PI 3 kinase.  Ref: 10407026 J Neurosci, 1999
- **CREB**  phosphorylation in activity deprived CN neurons is dependent on increased intracellular calcium resulting from influx through  **AMPA**  receptors and is mediated by calcium calmodulin dependent kinases and protein kinase A.  Ref: 10934277 J Neurosci, 2000
- Treatment of the tissue with a mixture of glutamatergic agonists N methyl D aspartate NMDA, amino methyl proprionic acid  **AMPA**  and kainate, all at 1 microM, or native glutamate 1 microM had no effect on the total  **CREB**  signal, but increased the pCREB signal, indicative of agonist stimulated phosphorylation of  **CREB**  on Ser133.  Ref: 9753174 Eur J Neurosci, 1998
- **AMPA**  receptor mediated, calcium dependent  **CREB**  phosphorylation in a subpopulation of auditory neurons surviving activity deprivation.  Ref: 10934277 J Neurosci, 2000
- Training in step down inhibitory avoidance 0.3 mA footshock is followed by biochemical changes in rat hippocampus that strongly suggest an involvement of quantitative changes in glutamate  **AMPA**  receptors, followed by changes in the dopamine D1 receptor cAMP protein kinase A PKA  **CREB**  P signalling pathway in memory consolidation.  Ref: 9361725 Braz J Med Biol Res, 1997

**Parallel relationship** (e.g. studied together, co-existance, homology, etc.)

- The results correlate with pharmacological findings showing an early post training involvement of  **AMPA**  receptors, and a late involvement of the D1 cAMP PKA  **CREB**  P pathway in memory consolidation of this task.  Ref: 9361725 Braz J Med Biol Res, 1997
- Involvement of hippocampal  **AMPA**  glutamate receptor changes and the cAMP protein kinase A  **CREB**  P signalling pathway in memory consolidation of an avoidance task in rats.  Ref: 9361725 Braz J Med Biol Res, 1997
- We found that neither  **AMPA**  kainate receptors nor NMDA receptors were able to stimulate independently a second messenger pathway that led to  **CREB**  phosphorylation or c fos gene expression.  Ref: 10414964 J Neurosci, 1999
